# Advances in the preclinical characterization of the antimicrobial peptide AS-48

**DOI:** 10.3389/fmicb.2023.1110360

**Published:** 2023-02-02

**Authors:** Rubén Cebrián, Marta Martínez-García, Matilde Fernández, Federico García, Manuel Martínez-Bueno, Eva Valdivia, Oscar P. Kuipers, Manuel Montalbán-López, Mercedes Maqueda

**Affiliations:** ^1^Department of Clinical Microbiology, Instituto de Investigación Biosanitaria Ibs.GRANADA, University Hospital San Cecilio, Granada, Spain; ^2^Department of Molecular Genetics, University of Groningen, Groningen, Netherlands; ^3^Department of Microbiology, University of Granada, Granada, Spain; ^4^Biomedicinal Research Network Center, Infectious Diseases (CIBERINFEC), Madrid, Spain

**Keywords:** antimicrobial peptide, bacteriocin, enterocin, AS-48, antimicrobial resistance

## Abstract

Antimicrobial resistance is a natural and inevitable phenomenon that constitutes a severe threat to global public health and economy. Innovative products, active against new targets and with no cross- or co-resistance with existing antibiotic classes, novel mechanisms of action, or multiple therapeutic targets are urgently required. For these reasons, antimicrobial peptides such as bacteriocins constitute a promising class of new antimicrobial drugs under investigation for clinical development. Here, we review the potential therapeutic use of AS-48, a head-to-tail cyclized cationic bacteriocin produced by *Enterococcus faecalis*. In the last few years, its potential against a wide range of human pathogens, including relevant bacterial pathogens and trypanosomatids, has been reported using *in vitro* tests and the mechanism of action has been investigated. AS-48 can create pores in the membrane of bacterial cells without the mediation of any specific receptor. However, this mechanism of action is different when susceptible parasites are studied and involves intracellular targets. Due to these novel mechanisms of action, AS-48 remains active against the antibiotic resistant strains tested. Remarkably, the effect of AS-48 against eukaryotic cell lines and in several animal models show little effect at the doses needed to inhibit susceptible species. The characteristics of this molecule such as low toxicity, microbicide activity, blood stability and activity, high stability at a wide range of temperatures or pH, resistance to proteases, and the receptor-independent effect make AS-48 unique to fight a broad range of microbial infections, including bacteria and some important parasites.

## Introduction

During the last century, different families of antibiotics and chemotherapeutics have been isolated/synthesized, characterized, and employed in the treatment of human and veterinary infections. Nowadays, antibiotic efficacy is seriously compromised because of several factors including over-prescription, misuse for non-bacterial infections, over-use in livestock and fish farming, poor infection control in hospitals, or poor hygiene and sanitation, among others. This has led to a moment in which the number of multidrug-resistant (MDR) bacteria is alarmingly increasing (Laxminarayan et al., 2013; McCullough et al., 2016). Antibiotic-resistant bacteria constitute one of the main threats to global health since they can affect anyone, of any age, in any country and lead to longer hospital stays, higher medical costs, and increased mortality (Hofer, 2019; Jit et al., 2020). With this background and according to the WHO, by 2050 MDR bacteria will be the first cause of death in the world, above conditions such as cancer or diabetes (O’Neill, 2014). Additionally, the number of new antibiotics approved for bacterial infection treatment is scarce in comparison with the necessities, and usually, they are chemical modifications of existing compounds, so they represent short-term solutions since the resistance mechanisms are already established in nature (Butler and Paterson, 2020). In the era of antimicrobial resistance development, new leads to fight MDR bacteria are urgently needed (Furuya and Lowy, 2006; Podolsky, 2018). In fact, 32 of the 50 antibiotics in the pipeline target bacteria included in the WHO priority list of pathogens that require urgent development of new treatments (WHO, 2017) but the majority of them have only limited benefits compared to antibiotics in use (WHO, 2022a). Last, investment in new antimicrobial development by the pharmaceutical industry is scarce (less than 5% in R&D) whereas the costs and time necessary for a proper development is extremely expensive and long (May, 2014; O’Neill, 2016).

“Re-discovering” old known molecules with antimicrobial activity to face the antibiotic crisis is an attractive alternative that is gaining the attention of the scientific community. In this sense, antimicrobial peptides (AMPs) are a promising alternative, by themselves or as adjuvants in combined therapy, for the treatment of MDR bacterial infections in both animals and humans (Cruz et al., 2014; Deslouches et al., 2020; Magana et al., 2020; Mookherjee et al., 2020). AMPs are a heterogeneous and ubiquitous group of peptides, posttranslationally modified or not, virtually produced by all the domains of life, that usually act as the first line in the defense against competitors and pathogens (innate immune response). The AMPs produced by bacteria are called bacteriocins. Nowadays, AMPs are pointed as an alternative to antibiotics because of their *in vitro* and *in vivo* potent activity against pathogens, their spectrum of activity (broad or narrow depending on the compound, so they can target specific bacteria reducing the effect over the microbiota), their usually low toxicity, and the ease to engineer them (Cotter et al., 2005, 2013; Montalbán-López et al., 2017, 2020b). In fact, nisin, the first bacteriocin described in 1928, unlike its coetaneous penicillin was relegated to the food industry as a food preservative due mainly to its proteinaceous nature (which raises concerns about the possible administration routes, stability or molecular size). This use has been broadly studied for other bacteriocins, especially those produced by lactic acid bacteria, which are common in the food industry and often present probiotic properties. In the last years, a renewed interest has appeared to study bacteriocins and AMPs to combat clinically relevant species, even though the costs of natural production and purification and/or synthesis, *in vivo* half-life and characterization of their toxicity/immunogenicity, pharmacokinetic-pharmacodynamics, and effect on the host microbiota remain as the main challenges for their development (Cotter et al., 2013; Theuretzbacher et al., 2020; Cesa-Luna et al., 2021).

Several classification schemes have been proposed for bacteriocins from lactic acid bacteria (Alvarez-Sieiro et al., 2016). Among them, lantibiotics and head-to-tail cyclized peptides show high potency against a broad range of microorganisms and have been studied in more detail. Both are ribosomally produced and posttranslationally modified peptides (RiPPs). The group of head-to-tail cyclized peptides (also referred to as circular bacteriocins in literature) is currently integrated by about 20 members (Vezina et al., 2020). They are characterized by the presence of a peptide bond between the N-and C-termini (Montalbán-López et al., 2011; Sánchez-Hidalgo et al., 2011; Dischinger et al., 2014). Head-to-tail cyclization is a rare posttranslational modification occurring in a handful of peptide families that confers them high stability against several physical, chemical, and biological conditions and makes them very interesting as scaffolds for drug design or by re-engineering linear peptides into cyclic ones, thereby increasing their stability and/or activity (Montalbán-López et al., 2012; Strijbis and Ploegh, 2014; Nguyen et al., 2015; Weidmann and Craik, 2016; Stevens et al., 2017).

The first circular bacteriocin (and peptide) discovered and also the best known to date from the genomic point of view to its applicability is the enterocin AS-48 (Gálvez et al., 1986). AS-48 is produced by several *Enterococcus* strains as a 105 amino acids precursor peptide, in which 35 amino acids correspond to the leader peptide that is cleaved before or concomitantly with the head-to-tail cyclization. The mechanisms underneath such a leader peptide cleavage and circularization remain unknown, both for AS-48 and for the rest of the circular bacteriocins described. The diversity in sequences, in leader peptides, or the absence of common motifs among these peptides suggest different ways to obtain the same results, the circular peptide backbone [this is further discussed in Sánchez-Hidalgo et al. (2011) and Gabrielsen et al. (2014)]. This review is focused on the state-of-the-art of clinical development of AS-48, discussing the importance of its mechanism of action reaching new and specific targets in bacteria and/or parasites, the broad spectrum of activity against several intra-and extracellular human pathogens, and its safety as a drug.

## AS-48 mechanism of action: Minimizing resistance development

**Figure 1:**
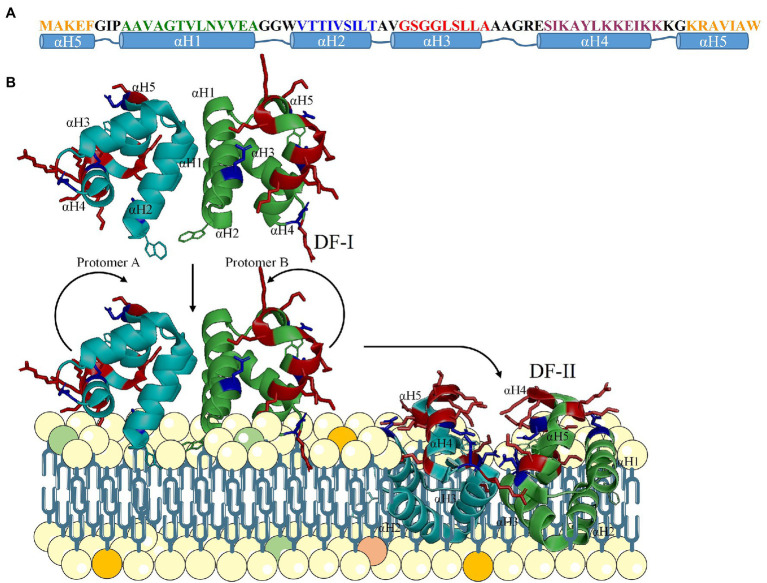
Secondary **(A)** and tertiary structure of AS-48 and proposed mode of action **(B)**. The water-soluble DF-I is attracted to the membrane due to electrostatic interactions and flips into the membrane-bound DF-II, that creates pores leading to cell death.

The main target of AS-48 is the cytoplasmic membrane of bacteria. Thus, Gram-positive bacteria are directly exposed to AS-48 whereas its activity is limited in the case of Gram-negative species because of the presence of the outer membrane that acts as a permeability barrier (Maqueda et al., 2004). Structurally, AS-48 is organized in 5 α-helices with a compact globular structure similar to saposin folding (Sánchez-Barrena et al., 2003; Figure 1A). It has a remarkable amphipathic surface where the positively charged residues (helices 4 and 5) are separated from the rest of the hydrophobic or uncharged residues (helices 1, 2, and 3) by a hypothetical plane delimitated by the Cα atoms of 4 glutamic acids (González et al., 2000; Sánchez-Hidalgo et al., 2008, 2011). This asymmetrical charge distribution, the stabilizing interaction of tryptophan residues, and the amphipathicity of the molecule play an essential role in the mechanism of action of the peptide (Cruz et al., 2021).

AS-48 molecules interact with each other in solution. In fact, in water solutions, AS-48 is organized as a dimer called DF-I in which the hydrophobic helices are sandwiched between two layers of cationic polar helices that are exposed to the solvent (Figure 1B; Sánchez-Barrena et al., 2003). In these conditions, the peptide presents a strong dipole moment that drives its approach to the bacterial membranes (Cebrián et al., 2015). At the membrane surface, the acidic pH destabilizes AS-48 DF-I dimer most likely because of the protonation of the glutamic acid side chains, promoting a rearrangement and transition from the water-soluble DF-I to the membrane embedded dimer DF-II (Figure 1B). In the latter, the polar helices are sandwiched in between the hydrophobic ones (exposed to the solvent/membrane). In this model, the protonated glutamic acid could recognize the phosphate group of phospholipids, and the positively charged and hydrophobic residues would interact with the polar head groups and the aliphatic chains of phospholipids, respectively (Sánchez-Barrena et al., 2003; Cebrián et al., 2015). The peptide is finally inserted in the membrane where it forms pores, abolishing the membrane potential, and cells lyse (Gálvez et al., 1991; González et al., 2000; Abriouel et al., 2001; Cruz et al., 2013). This mechanism of action is highly specific for bacterial cells due to the abundance of negatively charged phospholipids in their membrane and it is independent of their membrane potential or any receptor, so that even bacterial cells with low or without any metabolic activity may be targeted (Gálvez et al., 1991).

However, the bacterial lysis induced by AS-48 is not the only ultimate cell-death mechanism since in many cases no bacteriolysis has been observed (for instance, members of the Phylum *Actinomycetota* such as *Micrococcus, Mycobacterium*, *Nocardia*, *Cutibacterium, Gardnerella,* or the majority of the susceptible Gram-negative bacteria such as *Agrobacterium tumefaciens*, *Salmonella* spp., or *Escherichia coli*). Recently electronic scanning microscopy images of AS-48-treated *Staphylococcus aureus* showed that the cells remain together after the division septa formation, which suggests alternative unraveled mechanisms of action rather than solely membrane lysis (Velázquez-Suárez et al., 2021).

**Figure 2:**
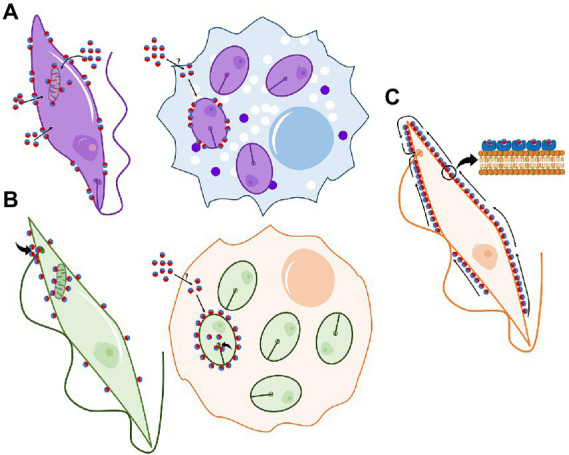
Schematic representation of AS-48 mechanisms of action on parasites. **(A)** Activity of AS-48 on *Leishmania* spp. promastigotes (extracellular) and amastigotes (inside macrophages). AS-48 induces pore formation in the cytoplasmic membrane that further grants AS-48 access to the cytoplasm reaching the mitochondria. **(B)** Activity of AS-48 on *T. cruzi* trypomastigotes/epimastigotes (extracelluar) and amastigotes (inside Vero cells). AS-48 is slowly endocytosed by the flagellar pocket and once inside the cells it reaches the mitochondria. **(C)** Activity of AS-48 on *T. brucei*. AS-48 binds VSG proteins on the parasite surface and it is rapidly internalized by the flagellar pocket inducing apoptosis.

Most bacteriocins have no effect on eukaryotic cell lines at the concentrations required to inhibit bacteria, which is a positive trait for a clinical application as antibacterial compounds. Surprisingly, and although AS-48 showed no activity against several eukaryotic organisms tested, like the yeast S*accharomyces* or the amoebas *Naegleria* spp. or *Acanthamoeba* spp. (Gálvez et al., 1989), parasites from the *Trypanosomatidae* family have been reported to be very susceptible (Abengózar et al., 2017; Martínez-García et al., 2018; Martín-Escolano et al., 2019). Interestingly, a deep inspection of the mechanism of action against these cells indicates that the activity involves different targets and mechanisms of action. Thus, two other different mechanisms of action have been proposed to explain the activity of AS-48 against these parasites. As commented before, the specificity of AS-48 to bacterial membranes is determined by the recognition of the negatively charged exposed membrane phospholipids, a feature present also in *Leishmania* (Abengózar et al., 2017; Figure 2A). However, the activity of AS-48 against this parasite cannot be explained just because of pore formation in the cytoplasmic membrane, since the leishmanicidal effect of AS-48 was not completed after 4 h of incubation, while the typical membrane permeation in bacteria is completed in a few minutes (Abengózar et al., 2017). The pores formed by AS-48 in *Leishmania* membrane are used as a gate to access the cytoplasm and, once inside, the peptide reaches the single mitochondrion, binding to the mitochondrial membrane and inducing a fast ATP depletion (Figure 2A). The damage in the functionality of the respiratory chain at the mitochondrion by AS-48 is also supported by the decrease in respiration and a rise in ROS production (Abengózar et al., 2017).

Amazingly, this mechanism is not shared against other trypanosomatids and, unlike *Leishmania*, pore formation in the membrane is not required against *Trypanosoma* spp. *Trypanosoma cruzi* is not permeated by AS-48. However, a similar mitochondrial dysfunction and ROS production were observed, which suggests that AS-48 may be endocytosed by this parasite across the flagellar pocket (Martín-Escolano et al., 2019; Figure 2B). Alterations in the mitochondrial functions were also related to other deleterious effects observed in this parasite such as the blocking of the glycolytic pathway and a decrease in the nucleic acid levels which is related to cell necrosis (Martín-Escolano et al., 2019; Figure 2B). Also, a killing mechanism unrelated to pore formation in the membrane has been described in *Trypanosoma brucei* (Martínez-García et al., 2018). Conversely to *T. cruzi*, in *T. brucei* the endocytic turnover of the surface components across the flagellar pocket is exceptionally high (Porto-Carreiro et al., 2000; Manna et al., 2014). This characteristic is involved in immune system evasion and therefore in the high resistance and survival of the parasite in the host and prevents the development of effective vaccines. However, it is the Achilles’ heel against AS-48. In fact, this parasite is the most susceptible pathogen to AS-48 described to date (approx. 1000 times more susceptible than the rest of the susceptible pathogens tested, including bacteria) (Martínez-García et al., 2018). In *T. brucei*, AS-48 enters *via* clathrin-mediated endocytosis after its interaction with the negatively charged variant surface glycoprotein (VSG; Figure 2C). The high number of VSG on the parasite surface (*ca.* 10^7^ molecules per cell) and its extremely rapid internalization and recycling by endocytosis (the VSG coat is completely internalized in 12.5 min) allow a fast accumulation of AS-48 inside the parasite. Once there, the bacteriocin can alter the normal structure of the membranous vesicles of the endocytic route inducing death by autophagy (Martínez-García et al., 2018). The parasiticidal activity of AS-48 against *T. brucei* is extremely fast compared to the other trypanosomatids tested, probably because of the prompt entering system (Martínez-García et al., 2018).

## Resistance to AS-48

As indicated above, AS-48 interacts with the lipids in the cell membrane and/or eukaryotic membranous organelles without the mediation of a receptor. Therefore, the development of stable resistance against this sort of membrane-active peptides is extremely difficult since it would require extensive modifications on a critical constituent of the cells that may drastically affect the cell physiology and viability. In fact, no resistance against AS-48 has been reported to the best of our knowledge.

Currently, four main mechanisms of adaptive resistance to bacterial AMPs have been described, namely peptide inactivation, surface remodeling, capsule production, or biofilm formation (Band and Weiss, 2015; Magana et al., 2020). So far, the only genetically encoded resistance to AS-48 is provided by the presence of the immunity protein (As-48D1) and the ABC-transporter (As-48EFGH) which are encoded by the *as-48* operon (Martínez-Bueno et al., 1998; Diaz et al., 2003). This transporter is also related to resistance to other similar bacteriocins (Teso-Pérez et al., 2021). Only AS-48 producer strains are fully resistant to the peptide, while the (heterologous) expression of them in other organisms provides partial levels of resistance (Martínez-Bueno et al., 1998; Fernández et al., 2007). Interestingly, cross-resistance to AS-48 was not observed even in the case of *Bacillus pumilus* B4107, which produces pumilarin, a very similar circular peptide that differs only in 10 amino acids compared to AS-48 (van Heel et al., 2017). Furthermore, the compact globular structure and cyclization confer high resistance to diverse proteases, which can provide longer half-life (Montalbán-López et al., 2008; del Castillo-Santaella et al., 2018).

In the case of other bacteriocins, resistance mechanisms are usually related to changes in bacterial outer layers (fluidity, lipid composition, the electrical potential of the membrane; cell wall thickness, and modification extent by, for instance, alanilation of teichoic acids, or a combination of all these factors) but also reducing bacteriocin accessibility to their receptors, downregulating the expression of genes involved in receptors or *via* proteolytic degradation of the bacteriocin (Soltani et al., 2021). Only a transient adaptive response to AS-48 has been detected in the case of *Listeria monocytogenes* at low temperatures and sub-minimum inhibitory concentrations (MIC) of AS-48. Adapted cells show increased resistance to other antimicrobials such as nisin or muramidases. This response is based in part on changes in the cell wall structure but specially on changes in the lipids of the cytoplasmic membrane, in which branched fatty acids and a higher C15:C17 ratio was determined. These adaptations disappear if AS-48 concentration is slightly increased or after repeated subcultivations in absence of the peptide (Mendoza et al., 1999).

## Antimicrobial activity of AS-48 against pathogenic bacteria

One of the most promising applications of AS-48 and other bacteriocins is related to their use as a food preservative (Cotter et al., 2005; Silva et al., 2018; O’Connor et al., 2020). In fact, some of them (or their producer strains) have been already approved as food additives by the FDA (Juturu and Wu, 2018). Moreover, enterococcal strains, even AS-48-producing strains, are usually isolated in many fermented products (Cebrián et al., 2012). For this reason, the activity of AS-48 alone or in combination with other antimicrobials as a food preservative against food-spoilage bacteria has been extensively analyzed (Abriouel et al., 2010; Ananou et al., 2014; Grande Burgos et al., 2014; Perales-Adan et al., 2018). Nevertheless, from a clinical point of view, the greatest risks in food safety come from the presence of foodborne pathogens. Bacteria are the most common cause of foodborne diseases. Foodborne illness occurs in two ways: when the pathogen is ingested with food and establishes itself in the human host, or when the bacteria produce toxins in the food that is ingested (Bintsis, 2017). A broad range of Gram-positive and Gram-negative bacteria such as *Clostridium botulinum, Clostridium perfringens, Clostridioides difficile, Bacillus subtilis, Bacillus cereus, Bacillus anthracis, Staphylococcus aureus*, *Listeria monocytogenes*, *Yersinia enterocolitica*, *Escherichia coli, Campylobacter jejuni, Salmonella* spp., *Shigella* spp., *Vibrio* spp., or *Brucella* spp. are mainly responsible for these diseases, which represent 3.6 million of infections per year just in the United States (Tauxe, 2002; Scallan et al., 2011; Martinović et al., 2016).

On the whole, AS-48 has shown potent activity *in vitro* against most of the Gram-positive bacteria tested including sporulated species as *B. cereus* (MIC 7.5–10 mg/l; Gálvez et al., 1989; Abriouel et al., 2002), although no direct effect on neither the spore viability nor germination initiation has been detected. However, just 10 min after germination, AS-48 was able to effectively kill the growing vegetative cell. Moreover, enterotoxin production by these bacteria was also inhibited by AS-48 (Abriouel et al., 2002; Grande et al., 2006). As an exception, in *Alicyclobacillus acidoterrestris* spore germination was abolished by AS-48 (Grande et al., 2005). Furthermore, a potent activity has been also observed against food-isolated *S. aureus* (MIC 1.14–5.21 mg/l; Caballero Gómez et al., 2013; Perales-Adan et al., 2018) or *L. monocytogenes* (MIC 0.1 mg/l), which is one of the most susceptible species (Mendoza et al., 1999). Because of the importance of *Listeria* as a foodborne pathogen, the effectivity of AS-48 alone or in combination with other antimicrobials or physical treatments against this bacterium (both planktonic or sessile) has been successfully determined in different food models such as ready-to-eat salads, raw fruits, vegetables, sausages or desserts (Gómez et al., 2012; Grande Burgos et al., 2014).

In the case of Gram-negative bacteria, the presence of the outer membrane makes them intrinsically more resistant to AS-48 (Abriouel et al., 1998). However, the combination of this bacteriocin with other treatments (physical or chemical) or with outer membrane disruptive agents has been evaluated in different food models as a good alternative to fight foodborne pathogens such as S*almonella enterica, E. coli* O157:H7, *Y. enterocolitica*, or *Shigella* spp. (Gálvez et al., 1989; Ananou et al., 2005; Cobo Molinos et al., 2008; Grande Burgos et al., 2014).

In addition to *in vitro* assays and in different foodstuff against food spoilage bacteria and foodborne pathogens, AS-48 has shown potent activity against a wide range of pathogens including both intra- and extracellular bacteria (Table 1). Most of them constitute a main concern in global public health and are discussed below. Remarkably, AS-48 has an intrinsic or potentiated bactericidal action in most of the cases that have been studied.

**Table 1:** Antimicrobial activity of AS-48 against pathogenic bacteria.

	Species/genus	ARS	Synergy	MIC mg/l	MIC μM
Gram-positive	***Enterococcus***	Yes	Lysozyme antibiotics HT	1.3–7.1	1.81–9.93
	***Staphylococcus***	Yes	Lysozyme HT	1.17–16.0	1.63–22.38
	***Mycobacterium***	Yes	Lysozyme antibiotics	2.0–64.0	2.79–89.51
	*C. acnes*	Yes	Lysozyme	0.62–2.5	0.86–3.49
Gram-negative	***K. pneumoniae***	No	HT^a^	>100	>139.87
	***P. aeruginosa***	No	HT^a^	>100	>139.87
	***E. coli***	Yes	HT^a^	>100	>139.87

Genera that are pointed as priority pathogens list for R&D of new antibiotics and specific research programs by the WHO are highlighted. For simplicity, MIC values indicate the concentration of AS-48 before using any synergistic treatment. ARS: antibiotic-resistant strains tested. HT: magnetic-induced hyperthermia. ^a^Necessary for sensitization to AS-48.

### Activity of AS-48 against the WHO priority list of bacteria

As part of its program in new antimicrobial research and development, the WHO published a list of pathogens for which new antimicrobials are urgently needed (WHO, 2017). Vancomycin-resistant *Enterococcus faecium* or vancomycin and/or methicillin-resistant *S. aureus* are among the bacteria for which new drugs are necessary with a high priority. Both species are intrinsically resistant to several antibiotics and can easily acquire resistance.

In general, enterococci are quite susceptible to AS-48 with MIC values in the low micromolar range. Recently, an enterococcal collection of clinical isolates with a wide range of antibiotics resistance (some of them resistant to 15/20 antibiotics tested) and virulence factors (some of them with 6/7 virulence factor genes) has been tested for its sensitivity to AS-48 (Montalbán-López et al., 2020a). The peptide is active at concentrations ranging between 1.3–7.1 mg/l (3.1 mg/l average) demonstrating an astonishing activity regardless of the virulence factors or antibiotic resistance displayed by the strains tested, including different vancomycin-resistant genotypes (Montalbán-López et al., 2020a). Currently, one of the most promising therapeutical strategies to fight MDR bacteria is based on the combination of two (or more) drugs to obtain a synergistic activity (Tyers and Wright, 2019). Synergism aims at the complete eradication of the infection, reducing the dosage of the drugs (and therefore the side effects) and the treatment timeframe. Also, the boosting effect of the combined treatment reduces the possibility to develop resistance by the bacteria when different mechanisms of action are simultaneously attacking the cell (Xu et al., 2018). For this reason, the combined effect of AS-48 with three well-characterized antibiotics commonly used against enterococci (namely vancomycin, gentamicin, and amoxicillin/clavulanate) has been tested. Interestingly, enterococcal strains that were highly resistant to these antibiotics (MICs >120 mg/l) are generally sensitized to them although not in all the cases, which suggests different resistance mechanisms to the combined treatment (Montalbán-López et al., 2020a).

Similarly, the sensitivity to AS-48 of clinically isolated *S. aureus* (*n* = 100) with a characterized resistance profile to 18 different antibiotics (33 strains were methicillin-resistant, MRSA) has been recently tested. All the strains were susceptible to AS-48 at concentrations ranging from 3 to 16 mg/l with an average resistance of 7.4 ± 0.46 mg/l and, as before, the activity of AS-48 was independent of the antibiotic-resistant profile (Velázquez-Suárez et al., 2021). Differences were neither observed between MRSA and methicillin-susceptible strains (Velázquez-Suárez et al., 2021). Remarkably, although *S. aureus* is intrinsically resistant to lysozyme, the combination of AS-48 with this muramidase significantly reduced the average MIC to 4.23 ± 0.43 mg/l (MIC ranging from 0.5 to 12 mg/l; Velázquez-Suárez et al., 2021). AS-48 was also active in established *S. aureus* biofilms, where important morphological changes were observed during the treatment, on both biofilm matrix structure (almost disappeared) and bacterial morphology (Velázquez-Suárez et al., 2021). Interestingly, clinically isolated *S. aureus* were 4.5 times more resistant to AS-48 than food-isolated *S. aureus*. The MIC of AS-48 against this species was ranging between 1.17 and 3.12 mg/l (average 1.63 mg/l), which suggests that no pathogenic factor could be related to higher AS-48 tolerance (Perales-Adan et al., 2018; Velázquez-Suárez et al., 2021). Besides, in the case of food-isolated *S. aureus*, an additive effect was observed for combinations with other compounds with different mechanisms of action such as the lantibiotic nisin (AS-48 MIC was reduced to 0.36 mg/l in the presence of 0.03 mg/l of nisin) which suggests the absence of cross-resistance for these two bacteriocins (Perales-Adan et al., 2018).

### Activity of AS-48 against *Actinomycetota* and *Mycobacterium*

Some species belonging to the phylum *Actinomycetota* (formerly *Actinobacteria*) are pathogens widely distributed worldwide. Acne is a chronic inflammation of the pilosebaceous unit and a recurrent skin condition affecting approximately 10% of the world’s population (Hay et al., 2014). The disease has a multifactorial etiology and is triggered initially during adolescence in susceptible individuals, but this condition can continue even in adults (especially in women). Although in general it occurs as a minor infection, in some cases, it can have severe psychological effects (Samuels et al., 2020). The relation of the commensal *Cutibacterium acnes* with the disease is still in question, but available data indicate that the presence of certain genotypes of this bacterium are directly implicated (Dréno et al., 2018). Besides acne, *C. acnes* is also related to other severe medical conditions linked to surgical procedures, foreign bodies, septicemia, and implant-associated infections (Achermann et al., 2014). AS-48 has shown potent activity against 20 clinical isolated *C. acnes* with a MIC between 0.62–2.5 mg/l (average MIC 1.22 mg/l) and this activity has been also achieved inside biofilms of this bacterium (Cebrián et al., 2018). This activity is enhanced by the combination of the peptide with lysozyme (an enzyme present in respiratory tract and ocular secretions as part of the body’s defense) commonly used in topical treatments, reducing the MIC of AS-48 to 0.5 mg/l. Remarkably, in this study, eradication of the bacterium was assessed at low concentrations. Unlike other bacteria, the killing kinetics for AS-48 against this species is quite slow (100% of killing effect after 48 h) and no lysis of the cell is observed (Cebrián et al., 2018).

More importantly, AS-48 is effective against mycobacteria, for which an additional specific programme has been developed by the WHO. Around 10 million people fall ill with tuberculosis worldwide each year and 1.5 million die, making tuberculosis one of the top 10 causes of death (WHO, 2022b). About one-quarter of the world’s population is estimated to be infected by *M. tuberculosis* but only 5–15% of these people develop the active disease. Tuberculosis infection is curable by a treatment using a combination of antimicrobial drugs for a long time due to the difficulties of the antibiotics to reach the infection sites of this slow-growing intracellular pathogen. Thus, a standard 6-months course of 4 antimicrobial drugs is commonly used. However, most of the people infected with tuberculosis live in low- and middle income countries, which makes access to this treatment difficult. In addition, the number of drug-resistant *M. tuberculosis* is rising and new drugs are urgently needed (WHO|Global Tuberculosis Report, 2019). Interestingly, AS-48 shows bactericidal activity against cultures of *M. tuberculosis* strains at concentrations between 8–64 mg/l without differences between actively replicating cells and the nonreplicative strains (Aguilar-Pérez et al., 2018). Other *Mycobacterium* species such as *M. xenopi* and *M. gordonae* (rarely related to human infections) are quite susceptible to AS-48 (MIC <2 mg/l; Aguilar-Pérez et al., 2018). The combination of AS-48 with lysozyme results in a potent synergistic interaction in all the cases tested. Thus, an astonishing MIC reduction against this bacterium (MIC <0.03 mg/l AS-48 in most cases) was observed (Aguilar-Pérez et al., 2018). Similar to the other actinobacteria tested, i.e., *C. acnes*, the killing kinetics is slower than against other bacterial phyla. Nevertheless, no bacteria are detectable after 6 days of treatment. Besides this, the antimicrobial activity of AS-48 in combination with the first-line antituberculosis drugs (ethambutol, isoniazid, streptomycin, and rifampin) proves an astonishing combined effect with ethambutol which suggests that the arabinogalactans wall may provide resistance against AS-48 in *Mycobacterium* spp. (Aguilar-Pérez et al., 2018). Next to the experiments against *M. tuberculosis* culture activity, the combination of AS-48 and lysozyme or ethambutol has been also tested against *M. tuberculosis*-infected macrophages. In the case of the combination with lysozyme, the synergistic effect is no longer observable but a surprisingly potent synergism was observed for the combination with ethambutol. In this case, a concentration as low as 2 mg/l of AS-48 and ethambutol caused bacterial death, whereas the MIC of each antimicrobial alone against this strain was 32 and 16 mg/l for AS-48 and ethambutol, respectively (Aguilar-Pérez et al., 2018).

### Boosting AS-48 activity and antimicrobial spectrum using magnetic nanocarriers

Often, bacterial infections take place in localized foci within the human body. The use of nanocarriers to deliver drugs to these points allows dose reduction and minimizes side effects. Magnetic nanoparticles are superparamagnetic nanocarriers that can be functionalized with diverse compounds depending on their surface characteristics. Those produced *in vitro* mimicking the magnetosome of *Magnetococcus marinus* are enriched in the peptide MamC. This makes the surface negative at physiological pH whereas it is neutral at acidic pH close to 5 (Valverde-Tercedor et al., 2015). Therefore, cationic compounds can be adsorbed at neutral pH and released under mildly acidic conditions such as those around the bacterial surface. Thus, AS-48 has been used to load biomimetic magnetic nanoparticles (BMNPs) and assayed against a broad range of bacteria. As expected AS-48-BMNPs are active against *S. aureus*, *E. faecalis*, and *E. faecium*, but also against the Gram-negative *E. coli*. Such a potent killing effect is not visible with the other Gram-negative species *Pseudomonas aeruginosa* or *Klebsiella pneumoniae* and could be related to the less acidic environment that they cause during growth compared to the former species (Jabalera et al., 2021).

BMNPs can be directed using a magnetic field and, under an alternating magnetic field, they rotate. When the field frequency is high enough, rotation causes a local temperature increase that facilitates drug release and probably a local mechanical damage in the external bacterial structures that potentiates the effect of AS-48 against *E. faecium*, *S. aureus*, *E. coli* but also *P. aeruginosa*, and *K. pneumoniae*. AS-48-BMNPs have been tested under such conditions, therefore, combining the inhibitory effect of AS-48 to that of the higher temperatures (45°C). The reduction in cell viability is already noticeable after just 15 min treatment and can achieve less than 0.5% survivors of *S. aureus* or *E. faecium* and less than 0.15% for the Gram-negative species mentioned above. Remarkably, avoparcin-resistant *E. faecium* and β–lactam-resistant *E. coli* remain susceptible to this treatment (Jabalera et al., 2021).

## Activity of AS-48 against parasites

Although AS-48 is not active against eukaryotic cells some parasites with a negatively charged surface could be susceptible to this bacteriocin. This is the case of *Trypanosomatidae* family, which encompasses several pathogenic species that cause serious diseases in humans and animals (Table 2; Pereira-Chioccola et al., 2000; Souto-Padrón, 2002).

**Table 2:** Antimicrobial activity of AS-48 against several members of *Trypanosomatidae* family.

Parasites	Developmental forms	IC_50_ (μM)	Source
*Leishmania donovani*	Promastigotes	3.9 ± 1.1	Abengózar et al. (2017)
*L. pifanoi*	Amastigotes	10.2 ± 1.2	
*Trypanosoma brucei brucei*	Bloodstream	0.0031 ± 0.0002	Martínez-García et al. (2018)
	Procyclic	0.14 ± 0.057	
*T. brucei gambiense*	Bloodstream	0.0026 ± 0.0001	
*T. brucei rhodesiense*	Bloodstream	0.0017 ± 0.0002	
*Trypanosoma cruzi* Arequipa	Epimastigote	0.76 ± 0.11	Martín-Escolano et al. (2019)
	Amastigote	0.99 ± 0.13	
	Trypomastigote	0.17 ± 0.04	
*T. cruzi* SN3	Epimastigote	1.16 ± 0.18	
	Amastigote	6.81 ± 0.89	
	Trypomastigote	0.11 ± 0.02	
*T. cruzi* Tulahuen	Epimastigote	0.82 ± 0.11	
	Amastigote	1.98 ± 0.22	
	Trypomastigote	0.19 ± 0.03	

### Activity of AS-48 against *Leishmania*

The first member from this family observed to be susceptible to AS-48 was *Leishmania* (Abengózar et al., 2017). Leishmaniases are a group of diseases (cutaneous leishmaniasis, visceral leishmaniasis, also known as kala-azar, and mucocutaneous leishmaniasis) caused by more than 20 *Leishmania* species and is transmitted by the bite of an infected sandfly insect vector. More than 1 billion people live in endemic areas for leishmaniasis (generally low-income countries) and more than 1 million new cases of leishmaniasis (mainly the cutaneous disease) are reported per year. The treatment is complex and should be administered by highly experienced health personnel. Besides, antileishmanial treatments cannot provide a cure, and the parasite remains in the human body causing relapses in case of immunosuppression. Also, some strains are already resistant to antiparasitic drugs (Ponte-Sucre et al., 2017). In their life cycle, the parasites are transmitted by the vector bite to the host as promastigotes that will be endocytosed by macrophages and other types o mononuclear phagocytic cells, and once inside the cell they will be transformed into intracellular amastigotes. In this form, they can multiply and proceed to infect other mononuclear phagocytic cells. When the vector takes the blood meal, the amastigotes change to promastigotes in the gut of the fly. The number is increased by division and then they migrate to the proboscis of the fly to infect a new host in the next blood meal (Bates, 2018). Thus, during its life cycle, both intracellular and extracellular forms are present, so drugs active against both parasite forms are desirable.

AS-48 can interact with *Leishmania* membranes. In the case of *Leishmania donovani,* the IC_50_ observed for promastigotes is 3.9 μM, while for *Leishmania pifanoi* axenic amastigotes it is 10.2 μM. Nevertheless, AS-48 can avoid the proliferation of the parasite at lower concentrations (1.3 and 7.5 μM, respectively; Abengózar et al., 2017). Remarkably, the leishmanicidal activity of AS-48 is not restricted to extracellular forms and 7 μM AS-48 reduces intracellular amastigotes parasitization index of the macrophage from 3.42 in untreated macrophages to 0.41 in the case of *L. pifanoi* (a reduction close to 90% after 48 h of treatment). Also, the percentage of infected macrophages decreases from 55.3% in untreated to 18.3% in AS-48 treated macrophages (Abengózar et al., 2017). These data suggest an intracellular activity of AS-48 in such phagocytic cells.

### Activity of AS-48 against *Trypanosoma cruzi*

Another parasite recently described to be susceptible to AS-48 is *Trypanosoma cruzi*, which causes American trypanosomiasis or Chagas disease. This is a parasitic infection affecting about 7 million people worldwide but mainly in endemic areas of 21 continental Latin American countries, where it is mostly transmitted by contact with feces or urine of infected triatomine bugs (Guhl, 2017). In the cycle of life different parasite forms are found. The infective one is the metacyclic trypomastigotes which are delivered in the feces of the bug and can enter inside the host across the bite of the vector, penetrating the cells of the area where they are transformed into amastigotes. Intracellular amastigotes can multiply and after that, they are delivered and transformed into trypomastigotes which are released into the blood and can infect other cells. When the bug bites, the trypomastigotes change to epimastigotes in the insect gut where they can multiply. Finally, they change to the infective metacyclic trypomastigotes that will be delivered into the feces (Onyekwelu, 2019). Although Chagas disease treatment is 100% effective if it is administered soon after the infection, it usually fails in the chronic phase, and the existence of drug-resistant strains hinders the disease treatment (Campos et al., 2014).

AS-48 has shown to be effective at least against three different strains of this parasite, with IC_50_ ranging between 0.76–1.16 μM for epimastigote forms, 0.11–0.19 μM for trypomastigote forms, and 0.99–6.81 μM for the intracellular amastigote forms (Martín-Escolano et al., 2019). Interestingly, the intracellular test was performed using infected Vero cells instead of phagocytic cells and the results support that AS-48 can enter inside the cells, killing the parasites without affecting the integrity of the Vero cells at the concentrations tested (Martín-Escolano et al., 2019).

Finally, the activity of AS-48 is comparable to marketed drugs such as benznidazole *in vivo* in an experimental mice model of Chagas (Martín-Escolano et al., 2020). In mice infection models, a notable reduction in the parasitemia level was observed when AS-48 was administered at 1 mg/kg while no effect was observed for benznidazole at the same concentrations (although a remarkable activity for this drug was observed when administrated at 100 mg/kg). AS-48 trypanocidal effect is potent from the beginning of the treatment, showing the lowest parasitemia subsequently and being undetectable at 34 days after the infection (Martín-Escolano et al., 2020). The reactivation of the parasitemia after immunosuppression in the chronic phase has been studied. In this case, a scarce (<10%) reactivation in the infection was noted in immunosuppressed mice treated with AS-48 compared to the benznidazole-treated mice (>50% of reactivation using 100 mg/kg). The presence of parasites was analyzed in 9 different parts of the mice (adipose tissue, bone marrow, brain, esophagus, heart, lung, muscle, spleen, and stomach) after the treatments. AS-48 is able to completely eliminate the parasites from bone marrow, brain, esophagus, lung, and spleen, while benznidazole at 100 mg/kg does the same in adipose tissue, brain, esophagus, spleen, and stomach (Martín-Escolano et al., 2020). These data show the potential of this AMP in the treatment of this infection.

### Activity of AS-48 against *Trypanosoma brucei*

The activity of AS-48 has been also tested against *Trypanosoma brucei* which is largely the most susceptible organism to the peptide. It is the etiological agent of African trypanosomiasis also called sleeping sickness which is transmitted by tsetse fly bites. This disease is distributed across 36 countries in sub-Saharan Africa with an estimation of 300.000 cases per year. This parasite causes big economic losses since nagana, the animal trypanosomiasis, is responsible for the inability to establish livestock in the high vector exposed areas (Sutherland and Tediosi, 2019). The treatment is based on old and toxic drugs and is dependent on the state of the disease. The drugs used in the first stage are safer and easier to administer than those required for the second stage when the parasite crosses the hematoencephalic barrier. Three different stages are found in the life cycle of *T. brucei*. Briefly, the infective metacyclic trypomastigotes transferred to the host by the fly bites are transformed into bloodstream trypomastigotes that can multiply in blood escaping from the immune response by the quick switching of the VSG. Unlike *T. cruzi*, *T. brucei* is always in the blood and no intracellular forms exist. When the fly ingests the trypomastigotes from the blood they change to procyclic trypomastigotes in the midgut of the insect and after that, they change to epimastigotes outside of the gut. Finally, they move to the salivary gland of the fly where they are transformed into infective metacyclic trypomastigotes (Matthews, 2005). AS-48 has shown astonishing activity *in vitro* against both, animal and human *Trypanosoma* parasites (*T. brucei brucei*, *T. brucei rhodesiense*, *T. brucei gambiense*), with IC_50_ against bloodstream trypomastigote forms ranging from 1.7 nM for *T. brucei rhodesiense* to 3.12 nM for *T. brucei brucei*, thus more than 1,000-fold reduction compared to other trypanosomatids or even the most susceptible bacteria. In the case of procyclic forms of *T. brucei brucei,* the IC_50_ is 140 nM. This difference could be related to the less endocytic activity of these forms and the importance of this *T. brucei* characteristic in the AS-48 action mechanism.

## Toxicity and safety of AS-48

Although bacteriocins are known since 100 years ago, their development and application are largely restricted to the food industry (Cotter et al., 2005). Nevertheless, the current emergency caused by MDR bacteria has given these compounds a renewed interest as new antimicrobial drugs (Cotter et al., 2013). Although their antimicrobial activity *in vitro* is well characterized, no or scarce data about hemolysis, toxicity on eukaryotic cell lines, or toxicity *in vivo* are usually provided, limiting their potential applications in a clinical scenario (Funck et al., 2020).

In the case of AS-48, preclinical characterization of its toxicity has been performed against a broad range of cell lines present in different organs, thus including breast (MCF10A) and colon (CCD18Co) epithelial cells (Cebrián et al., 2019), skin-related cells lines (A2058, CCD25sk; Cebrián et al., 2018), kidney (Vero; Martín-Escolano et al., 2019), immune system cell lines (MHS, J774.2, THP1, Raw264.7; Abengózar et al., 2017; Aguilar-Pérez et al., 2018), lung (MRC-5; Martínez-García et al., 2018) or liver (R1) cell lines (Baños et al., 2019a; Table 3). Similarly to many other cationic AMPs (Oddo and Hansen, 2017), AS-48 displays hemolytic activity against purified red cells. However, this activity disappears in the whole blood, while the antimicrobial activity is retained (Cebrián et al., 2019).

**Table 3:** AS-48 cytotoxicity against several eukaryotic cell lines.

Cell line	Source	IC_50_ (μM)	Source
MRC-5	Human lung fibroblasts	>12.5*	Martínez-García et al. (2018)
Vero	Monkey kidney	93.06 ± 5.67	Martín-Escolano et al. (2019)
CCD18Co	Human colon	>28*	Cebrián et al. (2019)
MCF10A	Human mammary gland	>28*	
A2058	Human skin	>28*	Cebrián et al. (2018)
CCD25sk	Human skin	>28*	
MHS	Murine macrophages	>18*	Aguilar-Pérez et al. (2018)
J774.2	Murine macrophages	>18*	
THP-1	Human monocytes	>18*	
Raw 264.7	Murine macrophages	>50*	Abengózar et al. (2017)
R1	Salmon liver	>14*	Baños et al. (2019a)

*Highest concentration tested.

In addition to cells, toxicity has been evaluated in animals. In the case of zebrafish embryos, no visible anomalies were observable at low doses of AS-48 after 48 h, but at concentrations higher than 3 μM several lethal effects were observed. Nevertheless, the absence of data for cationic AMPs toxicity studies in this model suggests that this may not be an appropriate model for these kinds of compounds (Cebrián et al., 2019). In the case of mice, topical and systemic applications of AS-48 have been tested. No adverse reactions have been observed when AS-48 was topically applied and neither any remarkable effect was observed in mice treated intraperitoneally with 5 mg/kg each 8 h during 2 days (Cebrián et al., 2019) or in rainbow trout exposed to 14 μM of AS-48 in baths for 96 h (Baños et al., 2019a). Recently, the subchronic toxicological potential of AS-48 when administrated in the diet has been also evaluated (Baños et al., 2019b). For that purpose, the mice were fed with 200 mg/kg of AS-48 for 90 days and no adverse effects were detected upon food and water intake, body weight, urine, and blood biochemical/hematological parameters. Besides, no alterations in the heart, spleen, thymus, kidneys, and small and large intestines were observed, and only small degenerative changes were observed in the liver. Interestingly, no other abnormal signs were found concerning liver function (including hematological and serum biochemistry tests; Baños et al., 2019b).

AS-48 has been also assayed for mutagenicicity and genotoxicity by means of the bacterial reverse-mutation assay in *Salmonella typhimurium* TA97A, TA98, TA100, TA102, TA1535 strains (Ames test) and the micronucleus test, respectively. The results obtained in both tests revealed no mutagenicity or genotoxicity (Cascajosa-Lira et al., 2020).

## Conclusion

Thirty-six years have passed since this unique circular bacteriocin was identified for the first time (Gálvez et al., 1986) and during this time it has been fully characterized from the genetical, structural, or biological activity points of view, being the circularization process the main challenge to be resolved yet (Sánchez-Hidalgo et al., 2011; Perez et al., 2018). The applicability of AS-48 has been mainly focused ever since on food preservation because of its potent antimicrobial activity against food spoilage and poisoning bacteria (Grande Burgos et al., 2014). However, the antimicrobial effect on human pathogens has favored numerous studies about toxicity and clinical application of AS-48 during the last few years. AS-48 shows no cross-resistance with other antibiotics and retains the activity against MDR bacteria. Moreover, it can sensitize resistant bacteria to antibiotics in certain synergistic combinations. Thus, the high potential of this peptide, alone or in combination with other drugs, for the treatment of several human (and animal) pathogens extends to multi-drug resistant strains. In fact, even activity against intracellular pathogens (bacteria and parasites) is documented. To the best of our knowledge, this kind of intracellular activity has not been reported for any other bacteriocin to date. An additional feature of AS-48 is its activity against biofilm-embedded bacteria (i.e., *S. aureus* and *C. acnes*) and slow-growing species, which often escape conventional treatments. Remarkably, membrane permeabilization and depolarization can be observed after just 24 h of treatment in the case of *Mycobacterium* spp., which shows the ability of this peptide to kill bacteria with very low metabolic activity. The preliminary studies using nanocarriers loaded with AS-48 open new scenarios to boost the activity against local infections caused by different bacteria, in which the ability to kill cells independently of their metabolic activity and growth rate can constitute a great advance. Preclinical toxicological analyses have shown that AS-48 is a safe molecule with high selectivity for bacteria and low toxicity towards human and animal cell lines at inhibitory concentrations. Its efficacy *in vivo* in mice models encourages us to consider AS-48 as a safe and efficient antimicrobial molecule.

Finally, and despite the effort in the preclinical characterization of AS-48, further analyses are still necessary to understand how AS-48 can enter inside the cell, killing intracellular pathogens, and to fully evaluate the toxicity using different administration routes, to discard an immune response against the peptide. Finally, further establishing the effectivity *in vivo* against additional infection models in both animals and humans is desired.

## Author contributions

RC, MM-G, and MM-L wrote the manuscript. RC and MM-G created the figures and tables. MF, FG, MM-B, EV, OK, and MM contributed to reviewing and editing the manuscript. All authors contributed to the article and approved the submitted version.

## Funding

MM-L acknowledges funding from FEDER Operational Program B-BIO-268-UGR20 and Proyectos de I + D + I, del Plan Andaluz de Investigación, Desarrollo e Innovación Grant P20_00339. RC acknowledges funding from the Instituto de Salud Carlos III (ISCIII, Miguel Servet program, ref: CP21/00113, Spain).

## Conflict of interest

The authors declare that the research was conducted in the absence of any commercial or financial relationships that could be construed as a potential conflict of interest.

## Publisher’s note

All claims expressed in this article are solely those of the authors and do not necessarily represent those of their affiliated organizations, or those of the publisher, the editors and the reviewers. Any product that may be evaluated in this article, or claim that may be made by its manufacturer, is not guaranteed or endorsed by the publisher.
